# Multidisciplinary approach to reducing falls for people with dementia on an older adult mental health ward

**DOI:** 10.1136/bmjoq-2025-003988

**Published:** 2026-06-01

**Authors:** Clarissa Sorlie, Gavin Shields, Tracy Connellan, Nathaniel Addo, Marco Aurelio

**Affiliations:** 1 East London NHS Foundation Trust, London, UK

**Keywords:** Dementia, Mental health, Quality improvement, Patient safety, Geriatrics

## Abstract

**Background:**

Falls can present a significant cost to individuals, their loved ones and the healthcare system. People with dementia on inpatient wards are at increased risk of falls, making this an important patient safety issue.

**Aim:**

The aim of the project was to reduce falls rate on an older adult mental health ward by 30% (from an average of 5.4 to 3.7 per 1000 occupied bed days) by September 2024.

**Methods:**

Using quality improvement methodology, a multidisciplinary team tested interventions to enhance therapeutic engagement, strengthen supportive nursing observations, mitigate medication-related risks and improve the ward environment.

**Results:**

The project surpassed its aim, reducing falls rate by 74% to 1.4 falls per 1000 occupied bed days.

**Conclusions:**

This project demonstrated the importance of a multidisciplinary approach to falls reduction, as well as the value of employing Plan-Do-Study-Act methodology for rapid testing and learning.

**WHAT IS ALREADY KNOWN ON THIS TOPIC**

- People with dementia are at risk of falls due to cognitive, environmental and medication-related factors, making multidisciplinary interventions essential to improve safety and reduce harm.

**WHAT THIS STUDY ADDS**

- This study supports a multidisciplinary bundle of interventions focused on enhancing therapeutic engagement, improving supportive nursing observations, managing medication-related falls risk and enhancing the ward environment.

**HOW THIS STUDY MIGHT AFFECT RESEARCH, PRACTICE OR POLICY**

- The findings support embedding multidisciplinary interventions on dementia wards to improve patient safety.

## Introduction

 Dementia is a group of related symptoms related to ongoing brain decline and can include memory loss, decline in thinking speed and challenges with everyday activities. The Alzheimer’s Society estimates there are currently 982 000 people in the UK with dementia; this figure is projected to rise to 1.4 million by 2040.^1^ People with dementia who are on inpatient wards are at increased risk of falls due to intersecting risk factors related to dementia, the hospital environment and prescribing.^2^ Dementia can be associated with disruption in spatial orientation, coordination challenges, motor dysfunction and impulsivity, all of which can contribute to an elevated falls risk.^3^ For people with dementia who are admitted to hospital, the inpatient environment can further contribute to falls through factors such as flooring, lighting and disorientation in an unfamiliar environment.^4^ Furthermore, Falls-Risk-Increasing drugs (such as antipsychotics and antidepressants) are commonly prescribed.^5^

Falls can significantly impact an individual’s social participation, functional ability and confidence.^6^ Individuals who have experienced a fall may develop fear of falling, and consequently restrict their activities of daily living, leading to a decline in their physical abilities.^7^ Falls while in hospital have been shown to increase length of stay.^8^ Falls also have a substantial financial impact on the National Health Service. According to an estimate by the National Institute for Health and Care Excellence, inpatient falls cost around £2600 per patient.^9^

In September 2022, Sally Sherman ward was identified as having the highest rate of falls in East London NHS Foundation Trust (ELFT). The ward is a 19-bedded mixed sex unit which provides care for older adults with cognitive impairments who require specialist nursing care to support with challenging behaviour. The multidisciplinary team (MDT) on the ward is made up of a consultant psychiatrist, general practitioner, specialist nursing team, pharmacist, support workers, occupational therapist, speech and language therapist, activity coordinator and dietician.

At this time, ELFT convened a large-scale quality improvement (QI) programme to improve observation practice on inpatient wards.^10^ The Sally Sherman team joined other inpatient teams across the Trust to share learning and scale ideas. The aim of the project was to reduce falls rate on a dementia ward by 30% (from an average of 5.4 to 3.7 per 1000 occupied bed days (OBDs)) by September 2024. This target of 30% was selected based on results from a national falls improvement collaborative across 19 National Health Service Trusts in 2017^11^ in which one ward achieved a 30% improvement. The improvement team determined this to be a realistic target.

ELFT provides mental health, community health and primary care services to a population of around 1.8 million people. Sally Sherman serves the population of Hackney, Tower Hamlets and Newham, where there are estimated 4221 people aged 65 and over with dementia.^12^

QI methods have been widely applied across UK healthcare settings to reduce falls. A QI project on a neuroscience ward in an acute NHS Trust in England demonstrated a 50% reduction in falls following the introduction of intentional rounding, where staff routinely check on patients at regular intervals.^13^ Similarly, QI work on an older adult ward identified several effective change ideas; however, only post-fall reviews led to sustained improvement.^14^

Across four Scottish mental health wards, evidence-based training for nursing staff reduced falls by 22%.^2^ A Welsh falls collaborative developed a multifactorial falls prevention care bundle across five wards, reinforcing the importance of multidisciplinary working.^15^

A systematic review found that structured interdisciplinary bedside rounds reduced falls by improving communication and decision-making,^4^ while an ethnographic study in England highlighted the importance of relational, person-centred interactions and recommended dedicated engagement support roles.^16^

## Methods

A balanced family of measures was developed including outcome, process and balancing measures. Outcome and process measures were displayed on statistical process control (SPC) charts to understand variation over time.^17^

The main outcome measure was falls rate per 1000 OBD on the ward each month. The falls rate was used to accommodate the variation in admissions each month. A fall was defined as an ‘event which results in a person coming to rest inadvertently on the ground or floor or other lower level’.^18^ Falls data were collected from the Trust’s incident reporting system and there was an average of 5.4 falls per 1000 OBD during the baseline period from March 2021 to December 2022. These data were displayed on a U Chart, a type of SPC chart which displays count data when the area of opportunity for an incident to occur varies.^17^

Two process measures were collected. The first process measure was the number of times an activity box was used each week. The second process measure was the percentage of supportive observations documented each week. This included all levels of supportive observations, including general, intermittent and continuous—whether within eyesight or arm’s length.^19^

The balancing measure was a retrospective analysis of the estimated reduction in cost of falls due to this work. The monetary value of a fall is difficult to calculate due to various patient factors. In this case an estimated average cost per fall in hospital of £2600 is used.^9^

A QI approach utilising the Model for Improvement^20^ provided a framework for this project, alongside a step-by-step sequence of improvement. The QI project team met for half an hour once a week to ensure steady progress. The team included nursing, therapy, pharmacy, medical and housekeeping staff. These multidisciplinary roles created a diverse set of perspectives on falls reduction. The project was coached by an Improvement Advisor and was sponsored by the directorate lead nurse.

Plan-Do-Study-Act (PDSA) cycles provided an iterative framework for evaluating the effectiveness of change ideas and assessing their impact^20^. This approach allowed the team to adjust their change ideas and build degree of belief before implementation. While each profession designed PDSA cycles to address specific challenges within their area of expertise, the team ensured alignment across disciplines through collaborative planning and discussions of observed outcomes at weekly project team meetings. The team reviewed their outcome measure data monthly to measure overall project progress.

**Figure 1:**
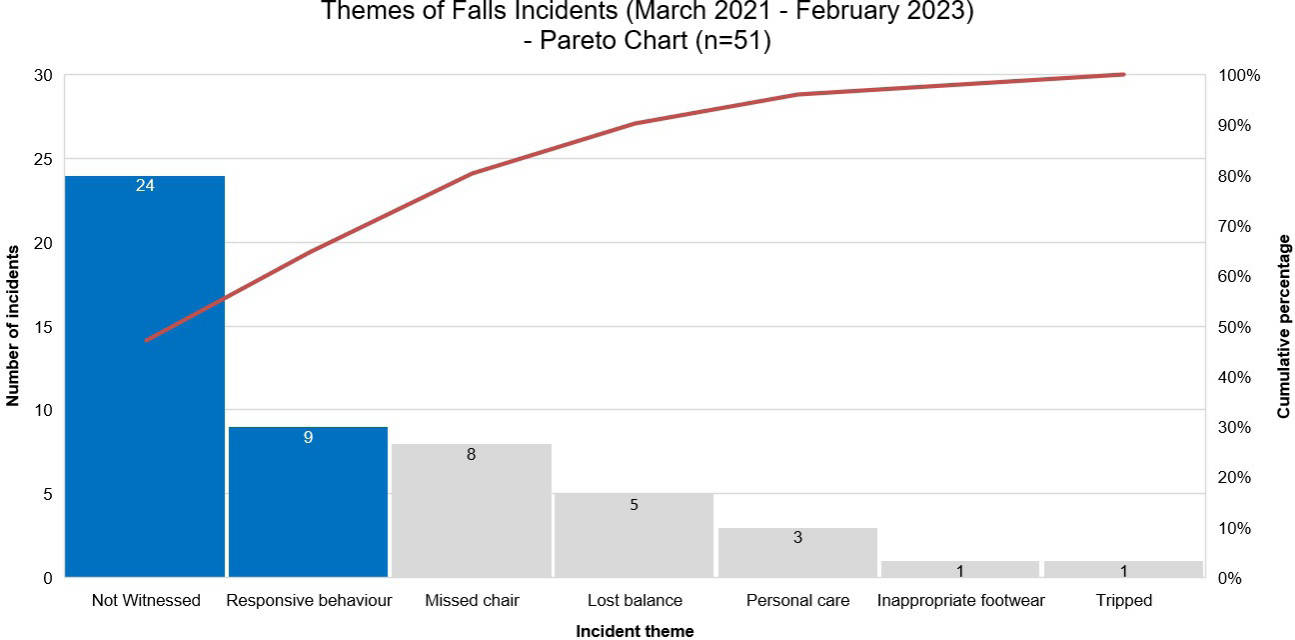
Pareto chart.

Two years’ worth of falls incident reports (51 incidents in total) were reviewed by the team and categorised into themes. Using these data, they created a Pareto chart, a tool which helps teams to focus their improvement efforts based on the Pareto Principle that 80% of problems are related to 20% of categories.^21^ This analysis provided the team with a foundation from which to develop targeted change ideas (see figure 1).

The Pareto chart highlighted that 47% of falls were not witnessed by staff. The next largest category (18%) of falls related to responsive behaviours—defined as disruptive or challenging actions that are a response to the environment and reveal underlying concerns (eg, pain).^22^

**Figure 2:**
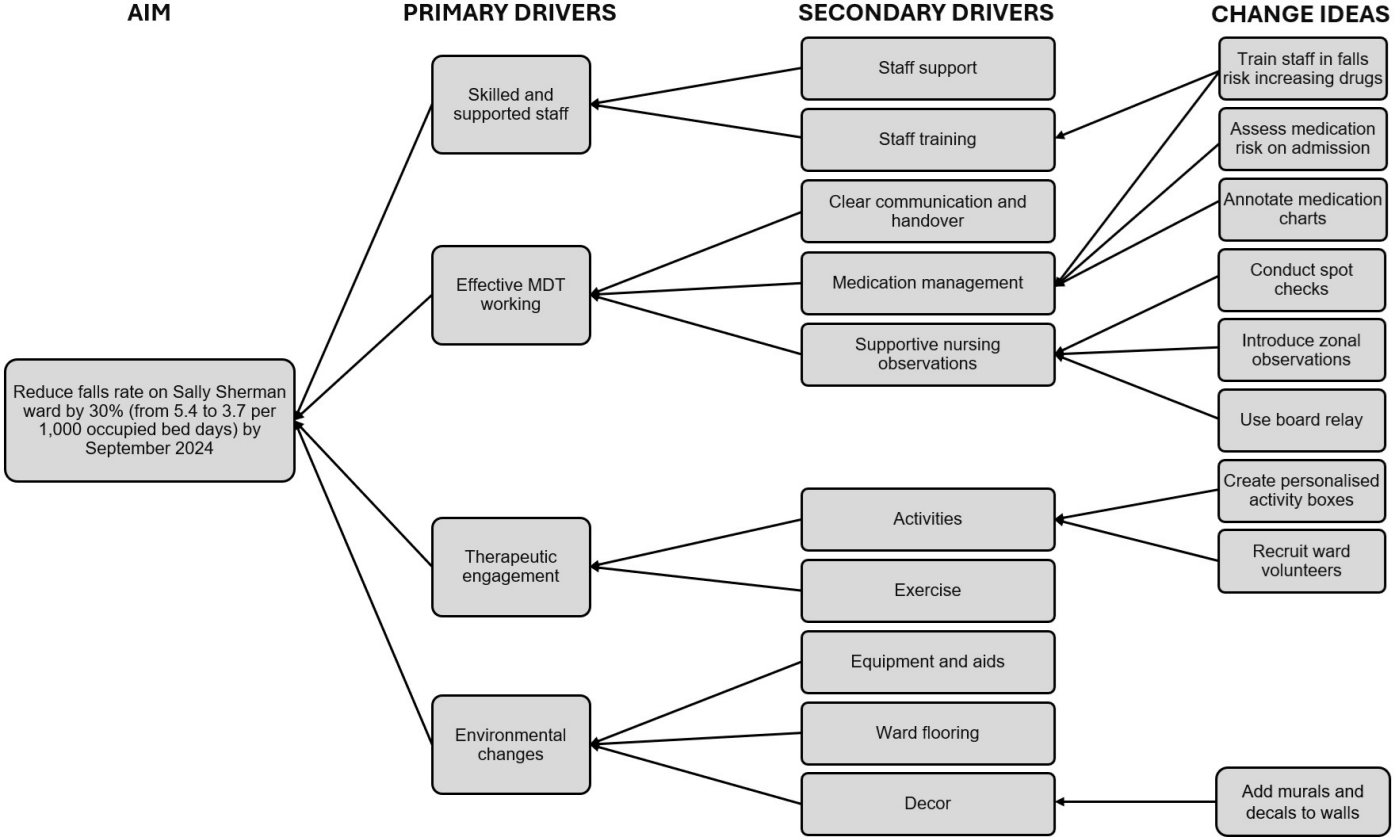
Driver diagram. MDT, multidisciplinary team.

After reviewing their baseline data and Pareto chart, the team generated change ideas on post-it notes. These ideas were grouped thematically and visually represented using a driver diagram.^23^ Four key areas were prioritised: improving therapeutic engagement, improving observation practice, developing medication reviews and changing the ward environment (see figure 2). Several change ideas were tested under each of these headings.

The occupational therapy team prioritised reminiscence work and developed personalised activity boxes for patients to reduce responsive behaviours and increase meaningful activity on the ward. The nursing team directed their efforts to improve the quality and reliability of nursing observations through spot checks and introduction of zonal observations. The pharmacy team and ward doctors collaborated to review medications contributing to falls risk, create posters listing medications which could contribute to falls and deliver staff training on medication risks. Meanwhile, the lead nurse enhanced the ward environment using murals of London landmarks to create places of interest and enrich the space for patients and their loved ones.

## Change idea 1: engagement activity boxes

Recognising that responsive behaviours were the second largest contributor to falls on the ward, the occupational therapy team theorised that increasing meaningful engagement in activity would improve patients’ mood and in turn reduce falls.

PDSA 1: The activities team (occupational therapists and activities coordinators) co-produced the ‘This is Me’ reminiscence workbook^24^ with families and carers to capture information about a patient’s background, significant life events and preferences.

PDSA 2 and 3: The activities team used the workbooks to develop and test individualised activity boxes, first with one patient and then a further two. Each box included printed instructions for use and a range of objects based on the patient’s interests, for example, a Bible, balls of yarn, and pictures of Diana, Princess of Wales. Staff used the objects and information in the boxes to engage patients in meaningful activity and conversation based on their interests. The team then gathered feedback from occupational therapy and activity staff about their utility. They prioritised use with patients on one-to-one observation and at risk of falls.

PDSA 4: The team demonstrated the activity boxes to the nursing team and gathered feedback on the perceived usefulness of the boxes, as well as discussing practical details such as optimal box sizes and storage location. They decided to store plastic 12-litre boxes with lids in the ward’s Activity Room.

PDSA 5 and 6: The team asked one member of nursing staff who attended the demonstration to test the activity box during one-to-one observations. They then expanded this test, asking additional nursing staff to test the boxes and provide feedback. Once nursing staff demonstrated confidence in using the activity boxes during one-to-one observations, responsibility for their use was transitioned to the nursing team. Nursing staff were asked to record each use, and the activity coordinator reviewed these data weekly, plotting it on a run chart, to determine the frequency and consistency of use.

PDSA 7: When it became clear that the boxes were not being used as frequently as intended, the team devised a new strategy: recruiting volunteers to embed consistent use of activity boxes. Over 6 months, 64 volunteers from university programmes in the local borough were recruited to use the boxes with patients during every shift, 7 days per week.

These volunteers received corporate and local inductions and were supported by the Trust volunteer team with timetabling and ongoing support. The volunteers also met with members of the MDT who offered further opportunities for learning. Volunteers developed meaningful relationships with patients, families, carers and the MDT and were offered further opportunities for learning by team members.

## Change idea 2: supportive observation audit checklist

The largest category (46%) on the Pareto chart related to falls that were not witnessed by staff and the team identified this as an opportunity to enhance the reliability of supportive observations that maintain contact between staff and patients and provide oversight of a patient’s location, safety and well-being during a shift.^19^

PDSA 1: The team tested a spot check audit to be completed every shift by the shift coordinator to learn whether observations were completed according to each patient’s care plan. The spot check was to be completed by the shift coordinator. The audit template incorporated the Trust observation record keeping audit, which evaluated records for completeness and accuracy.^19^ The team added a second section, which involved asking the member of staff undertaking continuous supportive observation of a patient what level of continuous observation (eyesight or arm’s length) the patient was on and the clinical rationale for the level of observation. When the team reviewed the audit documentation, they found that the audits were frequently missed and there were several questions on the form which were completed incorrectly.

PDSA 2: The team clarified the wording on the audit form to support understanding and shortened the form. Shift coordinators were again asked to test the form for a week. This time, the audit form was completed fully on every shift. This audit provided assurance that staff understood the reason and level of observation for each patient, with 100% of staff providing correct information. The audit process picked up several omissions in documentation, which were promptly addressed.

PDSA 3: The team continued to test the audit form for a further week and asked deputy ward managers to provide detailed feedback. Deputy ward managers reported that staff were more rigorous in their recording, knowing that spot checks would be completed at random. They found this particularly useful with staff who were new to the ward. They were satisfied with the content of the form and had no suggestions for improvement. The team agreed to implement this process on the ward, and added a prompt in the nursing handover document, to remind the person leading the handover to remind the team of the purpose of spot checks.

To further strengthen oversight of documentation gaps, the team added an additional process where the ward administrator checked observation records each morning, documenting the percentage of completion on a spreadsheet, and informing the ward manager and shift coordinator of any omissions. The percentage of completion of observations became a process measure for the project.

## Change idea 3: board relay

As part of the Trust-wide programme to improve observations, the team tested two change ideas that were scaled from other services.^10^ The first idea, board relay, was first tested on a female psychiatric intensive care ward. Based on the concept of a baton relay, the board on which one-to-one observations were documented was physically handed over to another member of staff without being put down.^25^ Nursing staff undertook observations for a period of 1 hour, after which the observation sheet was handed over to another nurse, with a verbal debrief

This idea was successful on a range of mental health wards in the Trust including Adult Mental Health, Child and Adolescent Mental Health and Forensics inpatient wards as evidenced by a reduction in missed observations. In the case of this ward, the intervention was tested briefly but quickly found to not be feasible. Staff consistently fed back that they always needed both hands free to be ready to quickly assist patients and were unable to put the board down during these instances for confidentiality reasons. Patients also attempted to take the board from staff. This idea was therefore abandoned.

## Change idea 4: zonal observation

The team then focused on zonal observations, which had been tested on a male psychiatric intensive care unit and scaled across the Trust.^10^ The idea presented an alternative method for observation, in which wards were designated into different zones with staff allocated per zone to continuously observe and engage with patients.

The Sally Sherman team adapted this idea to designate a ‘falls corridor’ for patients at high risk of falls, with allocated staff members at all times. Initially, this idea received limited buy-in from nursing staff as it presented a significant change in practice. The team believe that, if successfully embedded on the ward, this idea would reduce the number of staff allocated to one-to-one observations, thereby releasing staff to engage with patients. They are therefore keen to revisit this idea in the future, making incremental changes in response to their learning.

## Change idea 5: medication reviews

At the start of the project, most patients on the ward were prescribed multiple medications (polypharmacy), which is recognised in guidance from the National Institute for Health and Care Excellence as a significant risk factor for falls in older adults.^26^

The pharmacy team introduced a new process to assess falls risk related to medication on admission for every patient. These reviews were discussed during MDT meetings or ward rounds, allowing for timely deprescribing and medicines optimisation.

Another change idea involved annotating each patient’s electronic medication chart in the notes section to include their falls risk score and flag any medications known to increase fall risk. Counselling points such as ‘omit if patient appears drowsy’ were also included to mitigate fall risk. These annotations were made on admission and reviewed regularly during ward rounds.

Additionally, the pharmacy team delivered a presentation to nursing staff and the wider MDT, which aimed to raise awareness about medications commonly associated with falls. At the suggestion of the nursing team, the pharmacy team created a poster to display in clinical areas for ongoing awareness. Feedback from nursing staff was that this supported them to quickly identify patients at increased risks on such medication and prompt pharmacy for medication reviews where necessary.

## Change idea 6: improving the ward environment

The ward manager and lead nurse completed an audit of the physical environment of the ward, using the King’s Fund Enhancing the Healing Environment assessment tool.^27^ Conducting this audit involved walking around the ward area and considering how well the environment promotes seven areas: meaningful interaction; wellbeing; eating and drinking; mobility; continence and personal hygiene; orientation; and calm, safety and security. Flooring was identified as a particular area for improvement, as it did not meet the standards of being matte and non-reflective. The ward intends to replace the flooring as part of the life cycle.

The ward engaged a dementia environment specialist to help enhance the ward environment to promote orientation and engagement in meaningful activities. The dining room was transformed into a café, a post office façade was introduced, and the windows overlooking the ground floor were decorated with images of London. Decals were placed over the exit doors and decorated to look like walls to deter patients from trying to exit. Since introducing this change, patients have stopped pushing on the exit doors to attempt to leave the ward and this anecdotally reduced distress for service users.

At the end of the project, the team documented an implementation plan which included considerations such as standard work, documentation, staff education and training and monitoring the impact of changes. The ward manager and matron continued to monitor the monthly falls rate and completion of nursing observations as part of a quality control system.

## Results

**Figure 3:**
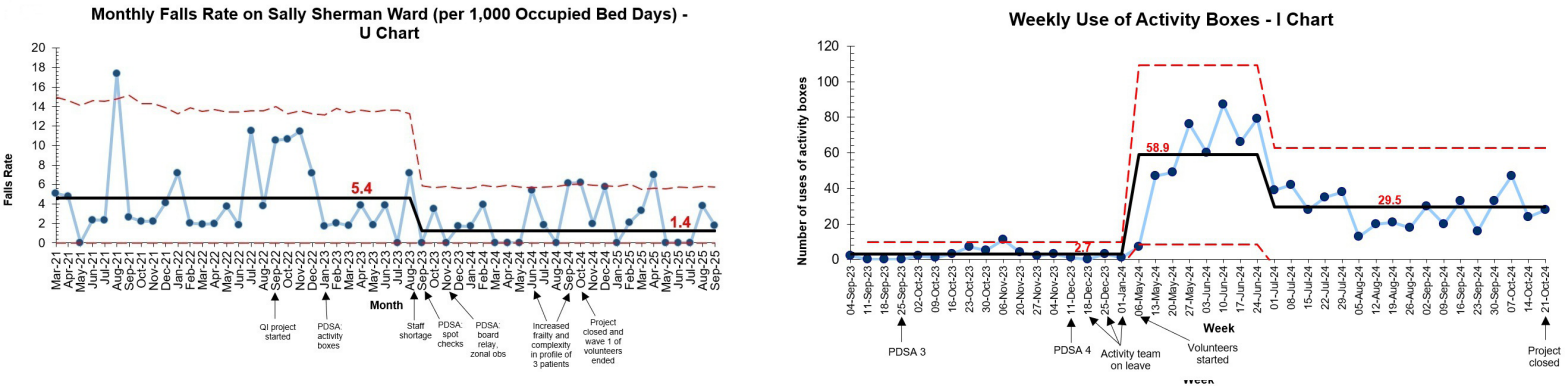
Monthly falls rate (U chart) and weekly use of activity boxes (I chart). PDSA, Plan-Do-Study-Act; QI, quality improvement.

Improvements over time were seen in all four measures used as part of this project (see figure 3).

Falls per 1000 OBDs fell by 74% from 5.4 per 1000 OBDs per month to 1.4 per 1000 OBDs per month. Results were sustained after the closure of the project (September 2024), with some months presenting higher falls rates related to increased frailty and complexity in the profile of three patients.

Before the recruitment of volunteers, personalised activity boxes were used an average of three times per week. The addition of volunteers significantly increased the use of activity boxes on the ward to an average of 59 times per week, enhancing patient engagement. Over time, as volunteers became more familiar with patients and their interests, their reliance on activity boxes declined to an average of 30 times per week. Engagement on the ward remained high, likely due to the strong relationships that had developed between volunteers and patients.

An average of 99.8% of nursing observations were completed correctly and these data were monitored daily by the ward administrator and the ward manager. This provided ward leadership with assurance of the robustness of documentation and allowed the ward manager and shift coordinator to quickly identify the rare instances where observations were not documented and promptly address any gaps with staff.

The balancing measure was the estimated cost of falls. Over the course of this work the estimated hypothetical cost avoidance is £33 600 per annum.

## Discussion

This QI project sought to reduce the number of falls on a dementia ward based in London. By using a systematic QI method, a team of staff and service users were able to use PDSA cycles to develop and test several change ideas that resulted in improvement. Over the course of the work, the rate of falls per 1000 OBD fell by 74% to 1.4 each month.

Several change ideas were tested, with changes observed in the data coinciding with testing of the activity boxes and a supportive observation checklist. Activity boxes were designed to improve therapeutic engagement on the ward. The importance of engagement on inpatient wards to prevent falls has been noted in other studies.^28 29^ Additionally, the activity boxes were co-designed, another feature which has been seen to help to reduce falls in inpatient settings.^16^ Over time, the impact of the activity boxes on relationships and therapeutic engagement became self-sustaining, leading to a natural decline in their use.

The use of checklists in inpatient settings to reduce falls has also been well noted, through providing a standardised way of understanding environmental risks and promoting consistent risk assessment.^30^ However, these need to be incorporated into daily practice to be truly effective. This supports learning from the work presented here, where the audit checklist incorporated the Trusts’ standard observation checklist. Additionally, checklists are considered most effective when paired with real-time feedback opportunities; with the checklist effectively becoming a tool to aid dialogue.^31^ In this instance, the checklist was incorporated into nursing handover huddles and provided a more structured way of feeding back risks.

Some change ideas that were part of a wider programme were tested in this ward but were found to be unhelpful in this context. The board relay and zonal observations proved challenging in these settings where staff needed to be free to assist service users. In the case of falls reduction, being able to assist service users to fall safely into an assisted fall has been seen to be an important factor in reducing harm from falls.^32^

A key strength of this project was the multidisciplinary nature of the project team. Each profession contributed its unique perspective and expertise on falls reduction, leading to a holistic view of the challenge. The importance of a multidisciplinary approach to falls reduction is supported by the findings of Macchiavello *et al*^15^ and Davey *et al*.^4^

Occupational therapists, physiotherapists and activity coordinators brought an appreciation for the importance of meaningful and purposeful activity to improving wellbeing and orientation. The success of the ward-based volunteer roles lends support to the recommendation from previous work^16^ for dedicated support roles on wards. Using PDSA methodology, results of small-scale tests were studied and adapted by the MDT until they had a high degree of belief that each change idea was ready to be implemented. This approach enabled the team to rapidly learn from tests of change, and from each other’s expertise.

Nursing staff brought expertise in risk assessment, care planning, person-centred care and supportive observation. A key contribution of this project has been establishing assurance processes for both the quality and documentation of supportive nursing observations, with clear and timely escalation routes for any omissions. The pharmacist and ward doctor provided an understanding of medication side effects and risks. While medication reviews and staff training have been recognised elsewhere as best practice,^15^ future work may benefit from evaluating the change idea to annotate patients’ medical charts with falls risk, including counselling points to mitigate falls risk.

Finally, the ward manager, lead nurse and dementia environmental specialist focused on the ward environment. Using a standardised environmental audit, also employed by similar projects,^15^ provided a useful structure for improving the ward environment.

Improvement has been sustained beyond the closure of the project and is monitored through quality control measures. The change ideas are being adopted across other older adult wards in the Trust. The change ideas tested were low cost, required limited staff training and were not limited by being used by specific staff groups, enhancing their potential replicability in other settings.

## Limitations

This work had several limitations. The lack of a control group and the time series design before and after limits the ability to attribute changes to the change ideas tested. Future work should adopt a control group design to build confidence in ideas. Second, we did not explore which combination of change ideas may have been most effective together. Experimental approaches such as planned experimentation can help understand which combinations of ideas result in the most effective improvement.^33^

Finally, the ward experienced significant absence from key members of ward leadership and the project team throughout the project lifecycle. This impacted on the team’s ability to maintain a predictable schedule of meetings, and meetings often took place ad hoc. This limited the feasibility of involving informal carers as full members of the project team, which would have further strengthened the diverse perspectives of the team.

## Conclusions

Using a QI approach to reducing falls, the team on Sally Sherman reduced the monthly falls rate from 5.4 to 1.4 per 1000 OBD. The introduction of volunteers on every shift across 7 days of the week and the use of personalised activity boxes had a significant impact on therapeutic engagement. Coupled with environmental changes to the ward, these initiatives helped make every day interesting and meaningful for patients. Falls risk was further reduced through medication reviews and robust nursing observations.

## Data Availability

All data relevant to the study are included in the article or uploaded as supplementary information.
